# British Orthopædic Society

**Published:** 1897-11-13

**Authors:** 


### BRITISH ORTHOPÆDIC SOCIETY.

### Spasmodic Wry Neck.

At a meeting of the society, held on November 5th, Mr. Noble Smith read a paper upon this subject. Out of 18 cases which he had treated, he had operated upon 11 by excision of a piece of the spinal accessory nerve, and in eight of these cases he had also had to operate upon the cervical nerves. In all the eight patients he had removed portions of the second, third, and fourth external branches of the posterior cervical nerves. In the three cases in which he had not performed this latter operation, two of the patients were for the present sufficiently contented with the results obtained by division of the spinal accessory alone. In the third case, although the sterno-mastoid was absolutely paralysed by the first operation, the patient said she did not realise any benefit, and could not be persuaded to submit to operation on the posterior cervical nerves; but it was stated that this patient had firmly made up her mind before anything was done that no treatment would relieve her. Mr. Noble Smith contended that, considering the almost uniform success of the double operation, this treatment could now be considered as a well-established and reliable method.

